# Mr. Simpson's Case of Prolapsus Uteri

**Published:** 1805-08-01

**Authors:** William Simpson

**Affiliations:** Surgeon. Workington


### *To the Editors of the Medical and Physical Journal.*

Gentlemen,

I Beg leave to transmit for your insertion a case of Prolapsus Uteri, in which the sponge pessary seems to have a decided and manifest superiority over all others. My reason for offering this observation to the medical world, is merely to corroborate the method recommended by Mr. Dawson (Vide Medical and Physical Journal, No. 72, page 129) whose valuable and well written paper I read with much pleasure.

About four months ago a case of prolapsus uteri came under my care; the lady informed me that she had been afflicted with this distressing complaint for some years; she had applied to a medical practitioner in the neighbourhood, who had recommended to her the common box-wood pessary, but that she still remained in a very uncomfortable situation; she therefore begged of me to think of some method by which she might attain more relief. I returned home without affording her much encouragement as to my success; however, at the time I was puzzling myself for some new contrivance, your very valuable Journal came to hand; hand; I confess I never felt more gratifyed than in the perusal of Mr. Dawson's excellent paper. I immediately had recourse to the method adopted by Mr. D. and I am happy to announce, with every possible advantage. The lady was almost overwhelmed with gratitude, and she can now go about her usual avocations with ease and pleasure.

I should certainly feel myself remiss were I not to return my sincere thanks to Mr. Dawson for his valuable information, and for laying it before the public.

I shall conclude with requesting every medical practitioner, when opportunity shall serve, to give the sponge pessary (agreeably to the plan adopted by Mr. Dawson) a fair trial, as standing in pre-eminence above all others.

I am, &c.

*Workington, July* 7, 1805. WILLIAM SIMPSON, Surgeon.

